# Rare manifestations and malignancies in tuberous sclerosis complex: findings from the TuberOus SClerosis registry to increAse disease awareness (TOSCA)

**DOI:** 10.1186/s13023-021-01917-y

**Published:** 2021-07-06

**Authors:** Matthias Sauter, Elena Belousova, Mirjana P. Benedik, Tom Carter, Vincent Cottin, Paolo Curatolo, Maria Dahlin, Lisa D’Amato, Guillaume B. d’Augères, Petrus J. de Vries, José C. Ferreira, Martha Feucht, Carla Fladrowski, Christoph Hertzberg, Sergiusz Jozwiak, John A. Lawson, Alfons Macaya, Ruben Marques, Rima Nabbout, Finbar O’Callaghan, Jiong Qin, Valentin Sander, Seema Shah, Yukitoshi Takahashi, Renaud Touraine, Sotiris Youroukos, Bernard Zonnenberg, Anna Jansen, J. Chris Kingswood, the TOSCA investigators, Nobuo Shinohara, Shigeo Horie, Masaya Kubota, Jun Tohyama, Katsumi Imai, Mari Kaneda, Hideo Kaneko, Yasushi Uchida, Tomoko Kirino, Shoichi Endo, Yoshikazu Inoue, Katsuhisa Uruno, Ayse Serdaroglu, Zuhal Yapici, Banu Anlar, Sakir Altunbasak, Olga Lvova, Oleg Valeryevich Belyaev, Oleg Agranovich, Elena Vladislavovna Levitina, Yulia Vladimirovna Maksimova, Antonina Karas, Yuwu Jiang, Liping Zou, Kaifeng Xu, Yushi Zhang, Guoming Luan, Yuqin Zhang, Yi Wang, Meiling Jin, Dingwei Ye, Weiping Liao, Liemin Zhou, Jie Liu, Jianxiang Liao, Bo YAN, Yanchun Deng, Li Jiang, Zhisheng Liu, Shaoping Huang, Hua Li, Kijoong Kim, Pei-Lung Chen, Hsiu-Fen Lee, Jeng-Dau Tsai, Ching-Shiang Chi, Chao-Ching Huang, Kate Riney, Deborah Yates, Patrick Kwan, Surachai Likasitwattanakul, Charcrin Nabangchang, Lunliya Thampratankul Krisnachai Chomtho, Kamornwan Katanyuwong, Somjit Sriudomkajorn, Jo Wilmshurst, Reeval Segel, Tal Gilboa, Michal Tzadok, Aviva Fattal- Valevski, Panagiotis Papathanasopoulos, Antigone Syrigou Papavasiliou, Stylianos Giannakodimos, Stylianos Gatzonis, Evangelos Pavlou, Meropi Tzoufi, A. M. H. Vergeer, Marc Dhooghe, Hélène Verhelst, Filip Roelens, Marie Cecile Nassogne, Pierre Defresne, Liesbeth De Waele, Patricia Leroy, Nathalie Demonceau, Benjamin Legros, Patrick Van Bogaert, Berten Ceulemans, Lina Dom, Pierre Castelnau, Anne De Saint Martin, Audrey Riquet, Mathieu Milh, Claude Cances, Jean-Michel Pedespan, Dorothee Ville, Agathe Roubertie, Stéphane Auvin, Patrick Berquin, Christian Richelme, Catherine Allaire, Sophie Gueden, Sylvie Nguyen The Tich, Bertrand Godet, Maria Luz Ruiz Falco Rojas, Jaume Campistol Planas, Antonio Martinez Bermejo, Patricia Smeyers Dura, Susana Roldan Aparicio, Maria Jesus Martinez Gonzalez, Javier Lopez Pison, Manuel Oscar Blanco Barca, Eduardo Lopez Laso, Olga Alonso Luengo, Francisco Javier Aguirre Rodriguez, Ignacio Malaga Dieguez, Ana Camacho Salas, Itxaso Marti Carrera, Eduardo Martinez Salcedo, Maria Eugenia Yoldi Petri, Ramon Cancho Candela, Ines da Conceicao Carrilho, Jose Pedro Vieira, José Paulo da Silva Oliveira Monteiro, Miguel Jorge Santos de Oliveira Ferreira Leao, Catarina Sofia Marceano Ribeiro Luis, Carla Pires Mendonca, Milda Endziniene, Jurgis Strautmanis, Inga Talvik, Maria Paola Canevini, Antonio Gambardella, Dario Pruna, Salvatore Buono, Elena Fontana, Bernardo Dalla Bernardina, Carmen Burloiu, Iuliu Stefan Bacos Cosma, Mihaela Adela Vintan, Laura Popescu, Karel Zitterbart, Jaroslava Payerova, Ladislav Bratsky, Zuzana Zilinska, Ursula Gruber-Sedlmayr, Matthias Baumann, Edda Haberlandt, Kevin Rostasy, Ekaterina Pataraia, Frances Elmslie, Clare Ann Johnston, Pamela Crawford, Peter Uldall, Paul Uvebrant, Olof Rask, Marit Bjoernvold, Eylert Brodtkorb, Andreas Sloerdahl, Ragnar Solhoff, Martine Sofie Gilje Jaatun, Marek Mandera, Elzbieta Janina Radzikowska, Mariusz Wysocki, Michael Fischereder, Gerhard Kurlemann, Bernd Wilken, Adelheid Wiemer-Kruel, Klemens Budde, Klaus Marquard, Markus Knuf, Andreas Hahn, Hans Hartmann, Andreas Merkenschlager, Regina Trollmann

**Affiliations:** 1 Klinikum Kempten, Klinikverbund Allgäu, Robert-Weixler-Str. 50, 87439 Kempten, Germany; 2 Research and Clinical Institute of Pediatrics, Pirogov Russian National Research Medical University, Moscow, Russian Federation; 3 SPS Pediatrična Klinika, Ljubljana, Slovenia; 4 TSA Tuberous Sclerosis Association, Nottingham, UK; 5 Hôpital Louis Pradel, Claude Bernard University Lyon 1, Lyon, France; 6 Tor Vergata University Hospital, Rome, Italy; 7 Karolinska University Hospital, Stockholm, Sweden; 8 Novartis Farma S.P.A, Origgio, Italy; 9 Association Sclérose Tubéreuse de Bourneville, Gradignan, France; 10 Division of Child and Adolescent Psychiatry, University of Cape Town, Cape Town, South Africa; 11 Centro Hospitalar Lisboa Ocidental, Lisbon, Portugal; 12 Universitätsklinik Für Kinder-Und Jugendheilkunde (Affiliated Partner of the ERN EpiCARE), Vienna, Austria; 13 Associazione Sclerosi Tuberosa ONLUS, Milan, Italy; 14 In Den Birken, European Tuberous Sclerosis Complex Association, Dattein, Germany; 15 Vivantes-Klinikum Neukölln, Berlin, Germany; 16 Department of Child Neurology, Medical University of Warsaw, Warsaw, Poland; 17 Department of Neurology and Epileptology, The Children’s Memorial Health Institute, Warsaw, Poland; 18 The Tuberous Sclerosis Multidisciplinary Management Clinic, Sydney Children’s Hospital, Randwick, NSW Australia; 19 Hospital Universitari Vall D’Hebron, Barcelona, Spain; 20 Institute of Biomedicine (IBIOMED), University of León, León, Spain; 21 Department of Pediatric Neurology, Necker Enfants Malades Hospital, Imagine Institute, Paris Descartes University, Paris, France; 22 Institute of Child Health, University College London, London, UK; 23 Department of Pediatrics, Peking University People’s Hospital, Beijing, China; 24 Tallinn Children Hospital, Tallinn, Estonia; 25 Novartis Healthcare Pvt. Ltd, Hyderabad, India; 26 National Epilepsy Center, Shizuoka Institute of Epilepsy and Neurological Disorders, Aoi-ku, UrushiyamaShizuoka, Japan; 27 Department of Genetics, CHU-Hôpital Nord, Saint Etienne, France; 28 St. Sophia Children’s Hospital, Athens, Greece; 29 University Medical Center, Utrecht, The Netherlands; 30 Pediatric Neurology Unit, Department of Pediatrics, UZ Brussel VUB, Brussels, Belgium; 31 Cardiology Clinical Academic Group, Molecular and Clinical Sciences Research Centre, St Georges University of London, London, UK

**Keywords:** Rare manifestation, Malignancy, TOSCA, TSC, Tuberous sclerosis complex

## Abstract

**Background:**

Tuberous sclerosis complex (TSC) is a rare multisystem autosomal dominant disorder caused by pathogenic variants in either the *TSC1* or *TSC2* gene. Common manifestations of TSC have been grouped into major and minor clinical diagnostic criteria and assessed in clinical routine workup. However, case studies point towards the existence of rare disease manifestations and to the potential association of TSC with malignant tumors. In this study we sought to characterize rare manifestations and malignancies using a large cohort of patients.

**Methods:**

TuberOus SClerosis registry to increAse disease awareness (TOSCA) is a multicenter, international disease registry collecting clinical manifestations and characteristics of patients with TSC, both retrospectively and prospectively. We report rates and characteristics of rare manifestations and malignancies in patients with TSC who had enrolled in the TOSCA registry. We also examined these manifestations by age, sex, and genotype (*TSC1* or *TSC2*).

**Results:**

Overall, 2211 patients with TSC were enrolled in the study. Rare manifestations were reported in 382 (17.3%) study participants and malignancies in 65 (2.9%). Of these rare manifestations, the most frequent were bone sclerotic foci (39.5%), scoliosis (23%), thyroid adenoma (5.5%), adrenal angiomyolipoma (4.5%), hemihypertrophy and pancreatic neuroendocrine tumors (pNET; both 3.1%). These rare manifestations were more commonly observed in adults than children (66.2% vs. 22.7%), in females versus males (58.4% vs. 41.6%; except for scoliosis: 48.9% vs. 51.1%), and in those with *TSC2* versus *TSC1* (67.0% vs. 21.1%; except for thyroid adenoma: 42.9% vs. 57.1%). In the 65 individuals with reported malignancies, the most common were renal cell carcinoma (47.7%), followed by breast (10.8%) and thyroid cancer (9.2%). Although malignancies were more common in adult patients, 26.1% were reported in children and 63.1% in individuals < 40 years. *TSC1* mutations were over-represented in individuals with malignancies compared to the overall TOSCA cohort (32.1% vs. 18.5%).

**Conclusion:**

Rare manifestations were observed in a significant proportion of individuals with TSC. We recommend further examination of rare manifestations in TSC. Collectively, malignancies were infrequent findings in our cohort. However, compared to the general population, malignant tumors occurred earlier in age and some tumor types were more common.

**Supplementary Information:**

The online version contains supplementary material available at 10.1186/s13023-021-01917-y.

## Introduction

Tuberous sclerosis complex (TSC) is a rare autosomal dominant disorder caused by pathogenic variations in the *TSC1* or *TSC2* gene. This results in hyperactivation of the mammalian/mechanistic target of rapamycin pathway, leading to hamartoma formation. The prevalence of TSC is estimated to be between 1/6800 and 1/15,000 and the incidence is estimated to be nearly 1:6000–10,000 live births [1–3]. It can affect all organ systems, leading to diverse clinical manifestations and has a broad variability, not only among individual patients but also within the affected families [1].

The 2012 International Tuberous Sclerosis Complex Consensus Conference provided recommendations to standardize the approach to manage this disorder. A system of major and minor criteria serves as a basis to establish the diagnosis clinically. Recommendations on surveillance and treatment mainly focus on these criteria [4]. However, the involvement of multiple organ systems at different stages of life presents a major challenge in the comprehensive clinical management of patients with TSC.

Over the last two decades, several clinical pathologies have been identified as associated with TSC, such as aortic and intracranial aneurysm [5, 6], arachnoid cysts [7, 8], lymphedema [9, 10], pancreatic endocrine tumor [11], pituitary adenoma [7], chordoma [12], and bone sclerotic foci [13]. Although these clinical pathologies are less frequent and not included as clinical diagnostic criteria, some of them can be life-threatening, while others may be challenging to integrate into a comprehensive clinical picture of the patients. Identification of these rare or less frequent manifestations and their clinical characteristics may help in their early diagnosis and contribute to ultimately preventing morbidity and mortality in patients with TSC.

A second important controversy in TSC is the fact that it remains a debate whether patients with TSC have an increased risk for malignant tumors. Until now, mostly renal cell carcinoma [14] has been identified in this context.

To the best of our knowledge, no systematic evaluation of these manifestations has been performed to date in a large patient cohort that might provide reliable results to aid clinicians in the management of TSC. Our aim is to present the rates and characteristics of various rare manifestations and malignancies observed in patients with TSC enrolled in the TuberOus SClerosis registry to increAse disease awareness (TOSCA) study and report differences in these rare manifestations and malignancies by sex, age, and* TSC* mutation.

## Materials and methods

### Study design, participants and data collection

A detailed methodology of the study has been published previously [15]. In brief, TOSCA is a multicenter, international disease registry structured to collect patient and disease information retrospectively and prospectively. It consists of a ‘core’ section and six ‘petals’ or ‘research projects’. In the core section of the study, information on the patient’s background, including demographics, familial and prenatal history, vital signs, and disease features, were collected at baseline and updated annually, wherever possible. Additional detailed information was collected in the six research projects that focused on subependymal giant cell astrocytoma, renal angiomyolipoma and lymphangiomyomatosis, genetics, TSC-associated neuropsychiatric disorders (TAND), epilepsy, and quality of life. Here, we present data on the rare manifestations, comorbidities and malignancies from the core section of the TOSCA registry.

Patients of any age with a documented visit for TSC in the preceding 12 months or newly diagnosed with TSC were enrolled into the TOSCA registry after obtaining written informed consent. Investigators collected data on rare manifestations by either selecting predefined items known to be associated with TSC or entering other items in a free text field.

For malignancies, data were collected for those patients who had either one or more of a number of predefined malignancies (renal, ovarian, testicular, and gastrointestinal malignancies). In addition, investigators could enter any other malignancy in a free text field.

### Assessments

Demographic and baseline characteristics between the patients with and without rare manifestations, and between patients with and without malignancies, were compared. For the purpose of this study, we defined rare manifestations as all manifestations declared as rare and disease-related by the investigators that did not fit under major or minor TSC clinical criteria as outlined in the 2012 Tuberous Sclerosis Consensus Conference recommendations and that were not a clear sequelae of major or minor manifestations (such as epilepsy).

We have prospectively categorized the rare manifestations by sex and mutation type, and by several syndromal complexes, such as tumors, malformations, vascular malformations, cystic lesions, endocrine disorders, and others (Table 2). Rare manifestations were also categorized into the following organ classes: vasculature, ear, nose and throat, endocrine system, eye, heart, gastrointestinal, liver, lymphatic tissue, nervous system, skeletal, urogenital, and others (Additional File 1: Table S1).

Rare manifestations reported by investigators in the free text field of the case report form that did not have an unambiguous description, were clearly not rare manifestations, or were typical manifestations of TSC (major/minor diagnostic criteria or epilepsy) were excluded from the analysis (Additional File 1: Table S2).

Malignancies observed in the TOSCA cohort were reported by organ. Rare manifestations and malignancies (either predefined or open field) were grouped based on organ system, and further by age, sex, and genotype. Available information on the age at first TSC diagnosis, mutation type and patient who received treatment for rare manifestation and malignancies are reported.

### Statistical analysis

All patients, without any major protocol deviation, enrolled in the TOSCA clinical study were included for analysis. SAS® Version 9.2 or later was used to perform all statistical analyses. Continuous variables were summarized with descriptive statistics (n, mean, standard deviation, range [minimum and maximum] and median). Frequency counts and the percentage of patients within each category were used for categorical data. Demographic and baseline characteristics between patients with and without rare manifestations and between patients with and without malignancies were compared using chi-square test for association and *Z*-test for means, as appropriate. A *p* value < 0.05 was considered statistically significant.

## Results

### Patient demographics and clinical characteristics

A total of 2211 patients with TSC were enrolled in the TOSCA registry. Of those, 382 (17.3%) individuals had rare manifestations and 65 (2.9%) had malignancies reported by investigators. Demographic characteristics in patients with and without rare manifestations and malignancies are shown in Table 1.

**Table 1:** Demographic characteristics in patients with and without rare manifestations and in patients with and without malignancies

Characteristics	Patients with rare manifestations, n (%)	Patients without rare manifestations, n (%)	*p* value	Patients with malignancies, n (%)	Patients without malignancies, n (%)	*p* value
**Age at consent, years**	N = 382	N = 1829		N = 65	N = 2146	
Mean (SD)	28.4 (16.71)	15.0 (14.15)	< 0.0001	32.7 (18.86)	16.9 (15.11)	< 0.0001
Median (range)	26.0 (0–71)	10.0 (0–71)		31 (0–68)	12 (0–71)	
**Age at first TSC diagnosis, years**						
Mean (SD)	12.4 (16.68)	5.8 (10.87)	< 0.0001	16.9 (18.94)	6.6 (11.95)	< 0.0001
Median (range)	2.0 (0–69)	1.0 (0–67)		10.5 (0–67)	1 (0–69)	
**Sex, n (%)**						
Male	159 (41.6)	900 (49.2)	0.007	22 (33.8)	1036 (48.3)	0.0214
Female	223 (58.4)	928 (50.8)		43 (66.2)	1109 (51.7)	
**Mutation, n (%)**						
*TSC1*	39 (21.1)	152 (18.4)	NS	10 (32.3)	181 (18.5)	0.0366
*TSC2*	124 (67.0)	525 (63.6)		15 (48.4)	634 (64.7)	
NMI	19 (10.3)	129 (15.6)		6 (19.4)	142 (14.5)	

NMI, no mutation identified; NS, not significant; SD, standard deviation; TSC, tuberous sclerosis complex

In patients with rare manifestations, TSC was diagnosed later than in those without (median: 2 years vs. 1 year; *p* < 0.0001). The female to male ratio was higher in patients with rare manifestations than in those without (female vs. male; 58.4% vs. 41.6% compared to 50.8% vs. 49.2%, respectively; *p* = 0.007). There was no difference in the mutation types (*TSC1*, *TSC2* or no mutation identified [NMI]) in participants with rare manifestations compared to individuals without (*p* = 0.687).

In patients with malignancies, TSC was diagnosed later compared to those without malignancies (median: 10.5 years vs. 1 year; *p* < 0.0001). We found a higher female to male ratio in patients with malignancies compared to individuals without malignancy (66.2%:33.8% vs. 51.7%:48.3%; *p* = 0.021). Considering differences in mutations, participants with malignancies had a significantly higher rate of *TSC1* mutations than participants without malignancies (*TSC1* vs. *TSC2* vs. NMI; 32.3% vs. 48.4% vs. 19.4% and 18.5% vs. 64.7% vs.14.5%, respectively; *p* = 0.037).

### Rare manifestations

In this study, 88 different manifestations were identified that were designated by the investigators as rare TSC-associated manifestations (Table 2). The five most frequent rare manifestations were bone sclerotic foci (39.5%), scoliosis (23%), thyroid adenoma (5.5%), adrenal angiomyolipoma (4.5%), hemihypertrophy and pNET (3.1% each) (Table 3).

**Table 2:** Rare manifestations observed in the study and grouped by tumors, malformations, endocrine disorders, cystic lesions, and others

Tumors (n = 248)	Malformations (n = 119)	Vascular malformations (n = 5)	Cystic lesions (n = 20)	Endocrine disorders (n = 6)	Others (n = 9)
Cartilaginous tumors of nasal septum (n = 1)	Calvarium sclerosis and thickening (n = 4)	Angiomatosis femoris (n = 1)	Liver cyst (n = 9)	Graves's disease (n = 2)	Laryngomalacia (n = 1)
Abdominal cystic pelvic tumor (n = 1)	Coloboma of iris (n = 1)	Aneurysm of anterior cerebral artery (n = 1)	Ovarian cyst (n = 8)	Hyperthyroidism (n = 2)	Leg edema (n = 1)
Adrenal angiomyolipoma (n = 17)	Club foot (n = 1)	Carotid aneurysm (n = 1)	Pancreatic cystic lesion (n = 2)	Hyperparathyroidism (n = 1)	Myositis ossificans (n = 1)
Angiomyolipoma of uterus (n = 1)	Congenital duodenal atresia (n = 1)	Chyloperitoneum (n = 1)	Pancreatic cystic lesion (small) (n = 1)	Hypothyroidism (n = 2)	Pyoderma gangrenosum (n = 1)
Angiomyolipoma other localization (n = 3)	Foot inversion (n = 1)	Chyloperitoneum in pelvis cavity (n = 1)	Perineural cyst (n = 1)	Hypopituitary [hypothyroidism] (n = 1)	Lymphedema (n = 7)
Bile duct adenoma (n = 1**)**	Anterior polar cataract (n = 1)	Lymphangioma in pelvis cavity (n = 1)	Arachnoid cyst (n = 2)		
Bladder lyoma (n = 1)	Hemihypertrophy (n = 12)				
Bone sclerotic foci (n = 151)	Hernia diaphragm (n = 1)				
Cardiac lipoma (n = 3)	Hip dysplasia (n = 2)				
Cartilaginous exostosis (n = 1)	Hyperostosis (n = 1)				
Chordoma (n = 3)	Hypospadias (n = 2)				
Chyloperitoneum lymphangioma (n = 1)	Anal malformation (n = 1)				
	Kyphosis (n = 1)				
Lymphangioma in pelvis cavity (n = 1)	Cerebellum angioma (n = 1)				
Dermoid cyst (n = 1)	Scoliosis (n = 88)				
Desmoplastic fibroma sinus maxillaris (n = 1)	Plantar fibromatosis (n = 1)				
Epidermal cyst in ear canal (n = 1)	Slipped femoral epiphyses (n = 1)				
Fibrolipoma (n = 1)	Spina bifida occulta (n = 1)				
Fibrous bone disorder (n = 1)	Syringomyelia (n = 1)				
Fibrous hamartoma T2 spine (n = 1)					
Gall bladder polyp (n = 3)					
Hamartoma breast (n = 1)					
Hamartoma stomach (n = 3)					
Intraductal papillary mucinous neoplasm (n = 1)					
Liver angiomyolipoma (n = 6)					
Liver hamartoma (n = 3)					
Liver hemangioma (n = 2)					
Lung tumor (n = 1)					
Lymph nodes from the renal resection region with a pattern of angiomyolipoma (n = 1)					
Neuroendocrinic carcinoid tumor grade 1 in the duodenum (n = 1)					
Neurofibroma (n = 1)					
Osteochondrome (n = 1)					
Ovarian tumor (n = 1)					
Pancreatic angiomyolipoma (n = 1)					
Pancreatic hamartoma (n = 1)					
Pancreatic tumor (n = 3)					
Parathyroid adenoma (n = 4)					
Parathyroid nodule (n = 1)					
Part solid and part cystic lesion in pancreas tail (n = 1)					
PEComa [perivascular epithelial cell tumor] of the uterus or ovary (n = 2)					
PEComa heart (n = 1)					
pNET (n = 12)					
Polypoid lesion in gall bladder (n = 2)					
Secreting pituitary adenoma (n = 3)					
Spleen angiomyolipoma (n = 9)					
Spleen hamartoma (n = 3)					
Stomach hamartoma (n = 3)					
Struma (n = 1)					
Submandibular gland tumor (n = 1)					
Thyroid adenoma (n = 21)					
Thyroid nodule (n = 2)					
Unspecified subcutaneous neoplasm in the thoracic region (n = 1)					
Unspecified tumor of nasal cavity (n = 1)					
Uterus myoma (n = 1)

**Table 3:** Demographics of patients with the ten most common rare manifestations by median age, sex, mutational status, and treatment

Rare manifestations	Overall, n (%) N = 2311	Patients with rare manifestation, n (%) N = 382	Median age at diagnosis, years, n (range)	Sex, n (%)			Mutation, n (%)			Patients who received treatment, n (%)
				Male (n = 159)	Female (n = 223)	*p* value	TSC1 (n = 39)	TSC2 (n = 124)	*p* value	
Bone sclerotic foci	151 (6.5)	151 (39.5)	31.5 (0–70)	59 (39.1)	92 (60.9)	0.0166	14 (24.1)	44 (75.9)	NS	3 (2.0)
Scoliosis	88 (3.8)	88 (23.0)	13 (0–50)	45 (51.1)	43 (48.9)	NS	6 (15.4)	33 (84.6)	NS	20 (22.7)
Thyroid adenoma	21 (0.9)	21 (5.5)	38.5 (12–69)	3 (14.3)	18 (85.7)	0.0015	4 (57.1)	3 (42.9)	NS	6 (28.6)
Adrenal angiomyolipoma	17 (0.7)	17 (4.5)	22.5 (3–45)	6 (35.3)	11 (64.7)	NS	0	3 (100)	NS	5 (29.4)
pNET	12 (0.5)	12 (3.1)	2111 (9–63)	5 (41.7)	7 (58.3)	NS	2 (28.6)	5 (71.4)	NS	8 (66.7)
Hemihypertrophy	12 (0.5)	12 (3.1)	4.5 (0–49)	5 (41.7)	7 (58.3)	NS	0	5 (100)	NS	1 (8.3)
Liver cysts	9 (0.4)	9 (2.4)	42 (14–60)	3 (33.3)	6 (66.7)	NS	1 (25.0)	3 (75.0)	NS	0
Spleen angiomyolipoma	9 (0.4)	9 (2.4)	13 (4–60)	4 (44.4)	5 (55.6)	NS	1 (20.0)	4 (80.0)	NS	3 (33.3)
Ovarian cysts	8 (0.3)	8 (2.1)	16 (9–31)	NA	8 (100)	0.0078	2 (33.3)	4 (66.7)	NS	1 (12.5)
Lymphedema	7 (0.3)	7 (1.8)	21 (0–48)	3 (42.9)	4 (57.1)	NS	1 (16.7)	5 (83.3)	NS	4 (57.1)
Liver angiomyolipoma	6 (0.3)	6 (1.6)	29 (16–53)	1 (16.7)	5 (83.3)	NS	0	3 (100)	NS	0

NS, not significant; pNET, pancreatic neuroendocrine tumor

Of the 382 patients with rare manifestations, tumors and cystic lesions were reported in 268 (tumors in 248 patients and cystic lesions in 20 patients), with female patients being more commonly affected than males (60.5% vs. 39.5%). Malformations were reported in 124 patients, equally affecting both sexes (male vs. female; 51.3% vs. 48.7%) and a majority of patients having *TSC2* mutation (85.2%). Malformations were observed at an earlier age (median age, 11 years), while tumors (median age, 28 years), cystic lesions (median age, 27 years), and endocrine disorders (median age, 31.5 years) were observed at a later age (Table 4).

**Table 4:** Malformations, tumors, and other manifestations in patients with rare manifestations

	Tumors and cystic lesions, n (%) n = 268				Malformations, n (%) n = 124				Others, n (%) n = 15			
	Tumors n = 248		Cystic lesions n = 20		Overall n = 119		Vascular malformations n = 5		Others* n = 9		Endocrine dysfunctions n = 6	
**Median age at diagnosis, years (range)**	28 (0–69)		27 (10–62)		11 (0–50)		24.5 (0–50)		9.5 (0–48)		31.5 (2–48)	
**Sex**	Male	Female	Male	Female	Male	Female	Male	Female	Male	Female	Male	Female
	98 (39.5)	150 (60.5)	4 (20)	16 (80)	61 (51.3)	58 (48.7)	1 (20.0)	4 (80.0)	4 (44.4)	5 (55.6)	3 (50)	3 (50)
**Mutation type**	*TSC1*	*TSC2*	*TSC1*	*TSC2*	*TSC1*	*TSC2*	*TSC1*	*TSC2*	*TSC1*	*TSC2*	*TSC1*	*TSC2*
	27 (26.2)	76 (73.8)	3 (23.1)	10 (76.9)	8 (14.8)	46 (85.2)	1 (100)	0	1 (14.3)	6 (85.7)	0	3 (100)
**Treatment**	40 (16.2)		1 (5.0)		26 (21.8)		3 (60.0)		5 (55.6)		4 (66.7)	

*Includes patients with laryngomalacia, lymphedema and myositis ossificans

As reported above, rare manifestations were predominant in female compared to male patients, except for scoliosis which was a little more frequent in male patients (51.1% vs. 48.9%; *p* = 0.733). Similar distribution patterns of *TSC* gene mutation were noted in patients with or without rare manifestation, i.e. rate of *TSC2* mutation being more than *TSC1*, with the exception of patients with thyroid adenoma who had a higher rate of *TSC1* mutations than *TSC2* (57.1% vs. 42.9%; p = 0.512). Upon stratification by age group, rare manifestations were more common in adult patients (> 18 years) (Table 5).

**Table 5:** Rate of the ten most common rare manifestations by age group

	Age, years n (%)						
	≤ 2 (n = 10)	2– ≤ 5 (n = 16)	> 5– ≤ 9 (n = 23)	> 9– ≤ 14 (n = 49)	> 14– ≤ 18 (n = 18)	> 18– ≤ 40 (n = 168)	> 40 (n = 98)
Bone sclerotic foci	0	0	1 (4.3)	5 (10.2)	5 (27.8)	88 (52.4)	52 (53.1)
Scoliosis	1 (10)	2 (12.5)	8 (34.8)	24 (48.9)	6 (33.3)	37 (22.0)	10 (10.2)
Thyroid adenoma	0	0	0	1 (2.0)	1 (5.6)	8 (4.8)	11 (11.2)
Adrenal angiomyolipoma	0	0	4 (17.4)	2 (4.1)	0	8 (4.8)	3 (3.1)
pNET	0	0	1 (4.3)	3 (6.1)	0	7 (4.2)	1 (1.0)
Hemihypertrophy	3 (30)	1 (6.3)	1 (4.3)	1 (2.0)	2 (11.1)	3 (1.8)	1 (1.0)
Liver cysts	0	0	0	1 (2.0)	0	3 (1.8)	5 (5.1)
Spleen angiomyolipoma	0	2 (12.5)	0	2 (4.1)	0	2 (1.2)	3 (3.1)
Ovarian cysts	0	0	1 (4.3)	4 (8.2)	0	3 (1.8)	0
Lymphedema	1 (10)	0	0	2 (4.1)	1 (5.6)	1 (0.6)	2 (2.0)
Liver angiomyolipoma	0	0	0	1 (2.0)	0	3 (1.8)	2 (2.0)

pNET, pancreatic neuroendocrine tumor

### Malignancies

Malignancies were reported in 65 patients. Most frequent malignancies observed were renal cell carcinoma (47.7%), breast cancer (10.8%), and thyroid cancer (9.2%).

Altogether, the percentage of female patients was significantly higher in the group of participants with malignancies (66.2%) compared to participants without malignancies (51.7%; *p* = 0.021) (Table 1). The predominance of females was consistent in patients with renal cell carcinoma (64.5%), but didn’t reach a statistical significance. In addition, the *TSC1*:*TSC2* ratio was significantly higher in patients with malignancies (*TSC1* vs. *TSC2* vs. NMI was 32.3% vs. 48.2% vs. 19.4%) compared to individuals without malignancies (18.5%:64.7%:14.5%; *p* = 0.037). Accordingly, the *TSC1:TSC2* ratio was markedly higher in patients with renal malignancy (36.4% vs. 53.3%) than in patients without malignancy (18.5% vs. 64.5%). Thyroid carcinoma (n = 6) was exclusively reported in six female patients, of which half had *TSC1* mutations (Table 6).

**Table 6:** Rate of malignancies in different organs in all patients, by sex and genotype

Organs	5-year prevalence of malignancies in general population (per 100, 000)^$, 25^			All, n (%) N = 65	Sex, n (%)			Mutation, n (%)		
	Overall	Male	Female		Male (n = 23)	Female (n = 42)	*p* value	*TSC1* (n = 11)	*TSC2* (n = 15)	*p* value
Kidney	13.4	16.5	10.3	31 (47.7)	11 (47.8)	20 (47.6)	NS	4 (36.4)	8 (53.3)	0.3176
Breast	181.8	–	181.8	7 (10.8)	0	7 (16.7)	0.0159	1 (9.1)	1 (6.7)	0.3961
Thyroid	26.2	11.2	41.4	6 (9.2)	0	6 (14.3)	0.0159	3 (27.3)	1 (6.7)	0.0104
Testis	7.4	7.4	–	5 (7.7)	5 (21.7)	0	0.0265	1 (9.1)	0	0.2230
Ovary	20.2	–	20.2	4 (6.2)	NA	4 (9.5)	NS	1 (9.1)	3 (20.0)	1.000
Bone, soft tissue	–	–	–	2 (3.1)	2 (8.7)	0	NS	0	1 (6.7)	1.000
Colon	62.8	67.4	58.0	2 (3.1)	0	2 (4.8)	NS	0	0	NE
Lung	27.9	34.1	21.6	2 (3.1)	0	2 (4.8)	NS	1 (9.1)	0	0.2230
Pancreas	3.7	3.9	3.5	2 (3.1)	2 (8.7)	0	NS	0	1 (6.7)	1.000
Brain (cerebral)	10.1	10.3	9.9	1 (1.5)	1 (4.3)	0	NS	0	0	NE
Eye	–	–	–	1 (1.5)	0	1 (2.4)	NS	0	0	NE
Liver	8.8	12.2	5.4	1 (1.5)	1 (4.3)	0	NS	0	0	NE
Skin*	12.7	13.1	12.2	1 (1.5)	1 (4.3)	0	0.4835	0	0	NE

*Melanoma of skin. ^$^5-year prevalence of malignancies have been presented for qualitative comparison between the general population and our cohort. NE, non-estimable; NS, not significant

Patients with malignancy appeared to be older (median 32.7 years) than those without (median 12 years; *p* < 0.0001). Overall, malignancies were more common in adult patients (≥ 18 years) compared with pediatric patients (< 18 years) (Table 7). However, it is important to state that nearly one-third of renal cell carcinoma cases, half of thyroid carcinomas, half of bone and soft tissue malignancies, one-fourth of ovarian malignancies, and all of the pancreatic malignancies occurred in children (≤ 14 years). Overall, 26% (n = 17) of malignancies were detected in patients < 18 years and 63.1% (n = 41) in those < 40 years (Table 7). Three patients (all female) had two different malignancies: one patient had ovarian and thyroid malignancies, the second patient had colon and breast malignancies, and the third patient had renal and breast malignancies. No patient died due to malignancies during the study.

**Table 7:** Malignancies by organ class and age groups

Organs	5-year prevalence of malignancies in general population by age group (per 100, 000)^$, 25^						Age (years), n (%)					
	0–9	10–14	15–19	20–39	> 40	≤ 2 (n = 1)	> 2– ≤ 5 (n = 3)	> 5– ≤ 9 (n = 3)	> 9– ≤ 14 (n = 8)	> 14– ≤ 18 (n = 2)	> 18– ≤ 40 (n = 24)	> 40 (n = 24)
Kidney	2.1	0.48	0.51	1.7	34.3	1 (100)	3 (100)	2 (66.7)	3 (37.5)	1 (50.0)	10 (41.7)	11 (45.8)
Breast	0.02	1.2	7.7	46.5	450.5	0	0	0	0	0	1 (4.2)	6 (25)
Thyroid	0.28	4.3	9.5	20.6	54.4	0	0	0	1 (12.5)	1 (50.0)	1 (4.2)	3 (12.5)
Testis	0.97	3.1	6.7	12.0	9.2	0	0	0	0	0	4 (16.7)	1 (4.2)
Ovary	0.62	2.1	3.9	9.5	45.7	0	0	0	1 (12.5)	0	2 (8.3)	1 (4.2)
Brain	3.2	3.4	3.6	5.2	20.7	0	0	0	1 (12.5)	0	0	0
Colon	0.06	0.82	1.7	4.7	170.1	0	0	0	0	0	0	2 (8.3)
Bone, soft tissue	–	–	–	–	–	0	0	1 (33.3)	0	0	0	1 (4.2)
Pancreas	0.01	0.10	0.26	0.47	9.9	0	0	0	2 (25.0)	0	0	0
Lung	0.06	0.34	0.77	1.8	75.9	0	0	0	0	0	1 (4.2)	1 (4.2)
Skin*	0.11	0.70	1.7	4.2	31.4	0	0	0	0	0	1 (4.2)	0
Eye	–	–	–	–	–	0	0	0	0	0	1 (4.2)	0
Liver	0.72	0.49	0.84	1.8	22.4	0	0	0	0	0	1 (4.2)	0

^*^Melanoma of skin. ^$^5-year prevalence of malignancies have been presented for qualitative comparison between the general population and our cohort

## Discussion

### Rare manifestations

The consensus clinical TSC diagnostic criteria include commonly presented TSC manifestations and the surveillance recommendations mainly focus on these manifestations [4]. However, there are numerous additional manifestations of TSC that are reported. They may occur quite frequently (for example, bone sclerotic foci), but may be systematically missed as they are not usually of clinical relevance. This large cohort study provides a clear estimate of the frequency of rare manifestations. Bone sclerotic foci might be mainly detected as a by-product of thoracic computed tomography scans, which are recommended for adult women affected by TSC only in order to screen for lymphangioleiomyomatosis (LAM) [16]. This might explain the clear female predominance of this manifestation in our study. Although of minor clinical relevance, it is important to know about this rather frequent ‘rare manifestation’ as bone sclerotic foci could be misinterpreted as bone metastasis or bone secondaries, which might result in unnecessary and potentially invasive assessments [4, 17] in spite being a common benign manifestation of TSC. Expert advice should be sought in case of doubt in these patients.

In addition, bone sclerotic lesions are discussed to differentiate TSC-associated LAM from sporadic LAM [16]. Given its frequency, one could argue for introducing bone sclerotic foci to the clinical diagnostic criteria.

The consensus guidelines further recommend excluding TSC-associated manifestations, such as bone cysts, endocrinopathies, vascular aneurysms, and gastrointestinal polyps, from routine evaluation unless coupled with clinical symptoms or history due to the insufficient evidence of benefit [1].

TSC is a multisystem disorder based on defects in tumor suppressor genes. We therefore hypothesized that there might be additional rare manifestations and asked investigators to document clinical signs considered as possible TSC-associated rare manifestations.

In total, 88 different rare manifestations were recorded in 17.3% of patients in this study. This shows the complexity of the disease and highlights the limitations of systematic evaluation and treatment of rare manifestations. Most rare manifestations were more common in female patients and those with *TSC2* mutations, which is in line with findings from previous literature on rare manifestations such as lymphedema and angiomyolipoma [10, 18]. Tumors and cystic lesions in a broad variety of organs seem to occur (or at least are detected) at higher ages. However, clinical significance seems limited in most cases, as treatment was reported for only 16% of tumors and 5% of cysts. It is worth noting that treatment rates differed markedly depending on organ system. Details are given in Additional File 1: Table S1.

A relevant number of rare manifestations were malformations (occurring at younger ages), of which scoliosis and hemihypertrophy were the most frequent. Diagnosis of musculoskeletal malformations can be performed easily via careful physical examination in most cases, which might contribute to detection at an earlier age. Whereas hemihypertrophy was clearly more frequent in our cohort compared to the overall population (1 in 86,000 live births), the rate of scoliosis did not differ from reports of adolescent idiopathic scoliosis (3.8% vs. 3.3%) [19, 20]. This might raise the question of whether scoliosis is a (rare) manifestation of TSC or a coincidental finding. Although causality cannot be proven by a registry study, it is worth mentioning that 22.7% of the scoliosis patients in our study required treatment (compared to 0.3% with adolescent idiopathic sclerosis). This possibly points toward a higher degree of severity or an underestimation of mild cases [21]. As previously reported, vascular malformations, including arterial aneurysms, can occur in patients but seem to be quite rare (n = 5 corresponding to 0.2% of the participants) (Table 5)**.** Therefore, our data supports the 2012 consensus recommendations on not to perform the routine evaluation given the sparse numbers. If routine imaging of the brain is performed, it seems justifiable to screen for blood vessel abnormalities as 75% of arterial aneurysms in our cohort were reported to occur in the extra- or intracranial brain-supporting vessels [4].

### Malignancies

The TSC consensus guidelines do not specifically comment on the surveillance of malignancies in patients with TSC [4]. It is still a matter of debate whether TSC patients have an increased risk of malignant tumors [14].

This is the first study to report malignancies in a large and multinational cohort of patients with TSC. No deaths were reported due to malignancy during the study. Although, the overall rate of malignancies in our study was higher than reported by Jozwiak et al. [22] (2.9% vs. 1.1%), but taken as a whole, still low. Notably, TOSCA participants, including the cohort of patients with malignancy, were markedly younger (median age 31 years) compared to the overall population. This might explain why several other tumor types were found to be predominant in this cohort compared to the overall population. The most frequent cancer entities in the overall population, such as lung, colorectal, and prostate cancer (in men), occur at older median ages. Only breast cancer, which is the most frequent cancer in the overall female population, was also a frequent cancer type seen in our cohort [23].

The most frequent type of malignancy in our cohort was renal cell carcinoma, affecting 1.4% (31 patients) of participants, majorly observed in pediatric and young adults (10 patients, ≤ 18 years). The rate of renal cell carcinoma considerably exceeds the prevalence in the overall population. Similar findings have been reported previously; Yang et al. (2014) referred to an overall incidence of 2% to 4% for renal malignancies [23] and Peron et al. (2016) reported an overall renal cell carcinoma rate of 2.1% [14]. TSC-associated renal malignancies have been reported to be more frequently observed in females, which is in contrast with the overall population where the rate of renal malignancy is higher in males (6.6 vs. 3.9 per 100,000) (2). We, too, observed a higher rate of renal malignancy in female patients (64.5%).

All six thyroid cancer cases in our study were observed in females only, which seems to be more frequent than in overall population (compare Table 6). Another frequent tumor type observed in our cohort was breast cancer (females only), which is, however, the most frequent cancer type in the overall population. Interestingly, patients with PTEN hamartoma tumor syndromes are at higher risk for both aforementioned tumor entities (cumulative cancer risk at age 70: 77% for female breast cancer and 38% for thyroid cancer) [25]. Analogical to the *TSC1* and *TSC2* complex, PTEN is another negative regulator of the mTOR pathway.

It is important to be aware of malignancies in patients with TSC, even at young ages. The occurrence of renal cell carcinoma in children has been reported previously [18] and was evident in our cohort as well. Malignancies were not limited to adult individuals with TSC; approximately 25% of malignancies in our study were observed in individuals younger than 18 years and 63.1% in participants younger than 40 years, similar to Peron et al. [14]. Given the overrepresentation of young patients in our study (63.3% were younger than 19 years and 89.5% were younger than 41 years at inclusion), overall malignancy rates in TSC patients might be underestimated as most malignant tumors occur at higher ages.

In this study, the genotype–phenotype correlations were difficult to establish due to the low number of patients with malignancies. We report 32.3% of patients with malignancy harboring *TSC1* mutations compared to only 18.5% of patients who did not have malignancy. A previous study similarly reported a predominance of *TSC1* mutations in patients with malignancies [14]. *TSC1* contributes to both tumor-suppressive and pro-metastatic action of the TGF-β-Smad pathway and works independently of *TSC2*, which is essential for cellular growth arrest and epithelial to mesenchymal transition [26]. This could be one possible reason for *TSC1* predominance in patients with malignancies in our study. However, further research will be necessary to elucidate the molecular mechanisms.

The evaluation of rare manifestations in this study had several limitations. First, a sign or symptom was recorded as a rare manifestation of TSC per the investigator’s definition. Second, no systematic screening for specific manifestations was performed in the study, except investigations performed in routine clinical practice. The first bears the risk of falsely collating a coincident symptom (especially if recorded infrequently) as a rare manifestation, and the second might result in underestimation of clinically asymptomatic manifestations. With respect to malignancies, investigators were not asked to provide histology findings and no central review of histology could be performed. The study was not designed to monitor malignancy treatments and their outcomes; hence we cannot provide further details. The required frequency of radiological tests to monitor cerebral and renal manifestations in TSC patients might introduce a lead time bias that may affect the conclusions of the study with respect to age at diagnosis. Also, the chance of long-term genetic damage caused by the radiological dose at relatively young ages might be a factor to consider. Although potentially affected by selection bias, it is of note that no study participant died from cancer during the study. Moreover, the TOSCA registry was not specifically designed for rare manifestations, hence the incidence of rare manifestations may be underreported.

## Conclusion

Rare manifestations occur in a relevant percentage of TSC patients. However, variability is high and further systematic evaluations are required to shape diagnostic and surveillance strategies. Malignancies affected about 2% of the participants in our study. Compared to the overall population, malignant tumors occurred earlier in age and were more common in females and participants with *TSC1* mutation.

## Supplementary Information

**Additional File 1. Supplementary Table 1**. Overall rare manifestations by organ class.** Supplementary Table 2**. Rare manifestations and the rationale for their exclusion from the analysis.

## Data Availability

Novartis supports the publication of scientifically rigorous analysis that is relevant to patient care, regardless of a positive or negative outcome. Qualified external researchers can request access to anonymized patient-level data, respecting patient informed consent, contacting study sponsor authors. The protocol can be accessed through EnCePP portal http://www.encepp.eu/ (EU PAS Register Number EUPAS3247).

## Abbreviations

LAM: Lymphangioleiomyomatosis
NMI: No Mutation Identified
TOSCA: TuberOus SClerosis registry to increAse Disease Awareness
TSC: Tuberous Sclerosis Complex

## References

[1] Curatolo P, Bombardieri R, Jozwiak S (2008). Tuberous sclerosis. Lancet.

[2] Sampson JR, Scahill SJ, Stephenson JB, Mann L, Connor JM (1989). Genetic aspects of tuberous sclerosis in the west of Scotland. J Med Genet.

[3] O'Callaghan FJ, Shiell AW, Osborne JP, Martyn CN (1998). Prevalence of tuberous sclerosis estimated by capture-recapture analysis. Lancet.

[4] Northrup H, Krueger DA, and on behalf of the International Tuberous Sclerosis Complex Consensus Group. International Tuberous Sclerosis Complex Consensus: Tuberous sclerosis complex diagnostic criteria update: recommendations of the 2012 International Tuberous Sclerosis Complex Consensus Conference. Pediatr Neurol, 2013;49(4):243–54. 10.1016/j.pediatrneurol.2013.08.001. 10.1016/j.pediatrneurol.2013.08.001 PMC4080684 24053982

[5] Salerno AE, Marsenic O, Meyers KE, Kaplan BS, Hellinger JC (2018). Vascular involvement in tuberous sclerosis. Pediatr Nephrol.

[6] Boronat S, Shaaya EA, Auladell M, Thiele EA, Caruso P (2014). Intracranial arteriopathy in tuberous sclerosis complex. J Child Neurol.

[7] Itoua B, Joubert E, Le Bras Y, Picot F, Gautier F, Wertel F (1999). What is it? Bourneville tuberous sclerosis associated with an arteriovenous malformation, a pituitary adenoma and 2 arachnoid cysts. J Radiol.

[8] Tatli M, Guzel A (2007). Bilateral temporal arachnoid cysts associated with tuberous sclerosis complex. J Child Neurol.

[9] Hoshiai S, Oguma E, Sato Y, Konishi T, Minami M (2015). Congenital focal lymphedema as a diagnostic clue to tuberous sclerosis complex: report of two cases diagnosed by ultrasound. Skeletal Radiol.

[10] Geffrey AL, Shinnick JE, Staley BA, Boronat S, Thiele EA (2014). Lymphedema in tuberous sclerosis complex. Am J Med Genet.

[11] Mortaji P, Morris KT, Samedi V, Eberhardt S, Ryan S (2018). Pancreatic neuroendocrine tumor in a patient with a TSC1 variant: case report and review of the literature. Fam Cancer.

[12] McMaster ML, Goldstein AM, Parry DM (2011). Clinical features distinguish childhood chordoma associated with tuberous sclerosis complex (TSC) from chordoma in the general paediatric population. J Med Genet.

[13] Roach ES, Smith M, Huttenlocher P, Bhat M, Alcorn D, Hawley L (1992). Report of the Diagnostic Criteria Committee of the National Tuberous Sclerosis Association. J Child Neurol.

[14] Peron A, Vignoli A, La Briola F, Volpi A, Montanari E, Morenghi E (2016). Do patients with tuberous sclerosis complex have an increased risk for malignancies?. Am J Med Genet.

[15] Kingswood JC, Bruzzi P, Curatolo P, de Vries PJ, Fladrowski C, Hertzberg C (2014). TOSCA - first international registry to address knowledge gaps in the natural history and management of tuberous sclerosis complex. Orphanet J Rare Dis.

[16] Avila NA, Dwyer AJ, Rabel A, Darling T, Hong CH, Moss J (2010). CT of sclerotic bone lesions: imaging features differentiating tuberous sclerosis complex with lymphangioleiomyomatosis from sporadic lymphangioleiomymatosis. Radiology.

[17] Pui MH, Kong HL, Choo HF (1996). Bone changes in tuberous sclerosis mimicking metastases. Austral Radiol.

[18] Krueger DA, Northrup H (2013). International tuberous sclerosis complex consensus: tuberous sclerosis complex surveillance and management: recommendations of the 2012 International Tuberous Sclerosis Complex Consensus Conference. Pediatr Neurol.

[19] Tomooka Y, Onitsuka H, Goya T, Hayashida Y, Kuroiwa T, Kudo S (1988). Congenital hemihypertrophy with adrenal adenoma and medullary sponge kidney. Br J Radiol.

[20] Gore DR, Passehl R, Sepic S, Dalton A (1981). Scoliosis screening: results of a community project. Pediatrics.

[21] Scheral SA. Adolescent idiopathic scoliosis: Clinical features, evaluation, and diagnosis. https://www.uptodate.com/contents/adolescent-idiopathic-scoliosis-clinical-features-evaluation-and-diagnosis. Accessed 02 Nov 2020.

[22] Jozwiak S, Sadowski K, Borkowska J, Domanska-Pakiela D, Chmielewski D, Jurkiewicz E (2018). Liver angiomyolipomas in tuberous sclerosis complex-their incidence and course. Pediatr Neurol.

[23] Yang P, Cornejo KM, Sadow PM, Cheng L, Wang M, Xiao Y (2014). Renal cell carcinoma in tuberous sclerosis complex. Am J Surg Pathol.

[24] Kingswood JC, d’Augères GB, Belousova E, Ferreira JC, Carter T, Castellana R (2017). TuberOus SClerosis registry to increase disease Awareness (TOSCA)–baseline data on 2093 patients. Orphanet J Rare Dis.

[25] Bubien V, Bonnet F, Brouste V, Hoppe S, Barouk-Simonet E, David A (2013). High cumulative risks of cancer in patients with PTEN hamartoma tumour syndrome. J Med Genet.

[26] Thien A, Prentzell MT, Holzwarth B, Kläsener K, Kuper I, Boehlke C (2015). TSC1 activates TGF-β-Smad2/3 signaling in growth arrest and epithelial-to-mesenchymal transition. Dev Cell.

