# Supplementary material for: Rare manifestations and malignancies in tuberous sclerosis complex: findings from the TuberOus SClerosis registry to increAse disease awareness (TOSCA)
Source: Orphanet J Rare Dis. 2021 Jul 6;16:301. doi: 10.1186/s13023-021-01917-y (PMC8259106; doi:10.1186/s13023-021-01917-y)
Supplement: Supplementary file 1 — Additional File 1. Supplementary Table 1. Overall rare manifestations by organ class. Supplementary Table 2. Rare manifestations and the rationale for their exclusion from the analysis. [file 13023_2021_1917_MOESM1_ESM.docx]

**8 Appendix**

**Supplementary Table 1. Overall rare manifestations by organ class**

| Organs | All, n (%)  N=382 | Median age at rare manifestation diagnosis (years) | Sex, n (%) | | Mutation | | Treatment (Yes) |
| --- | --- | --- | --- | --- | --- | --- | --- |
|  |  |  | **Male**  **(n=159)** | **Female**  **(n=223)** | ***TSC1***  **(n=39)** | ***TSC2***  **(n=124)** |  |
| Blood vessels | 4 (1.0) | 24.5 | 1 (0.6) | 3 (1.3) | 1 (2.6) | 0 | 2 (50.0) |
| Aneurysm of anterior cerebral artery | 1 (25.0) | 50**^a^** | 0 | 1 (33.33) | 1 (100) | 0 | 1 (50.0) |
| Angiomatosis femoris | 1 (25.0) | ≤1**^a^** | 0 | 1 (33.33) | 0 | 0 | 0 |
| Carotid aneurysm | 1 (25.0) | 47**^a^** | 0 | 1 (33.33) | 0 | 0 | 1 (50.0) |
| Cerebellum angioma | 1 (25.0) | 2**^a^** | 1 (100) | 0 | 0 | 0 | 0 |
| Ear, nose, throat | 6 (1.6) | 2.0 | 5 (3.1) | 1 (0.4) | 1(2.6) |  | 5 (83.3) |
| Cartilaginous tumor of nasal septum | 1 (16.7) | ≤1**^a^** | 0 | 1 (100) | 0 | 0 | 1 (25.0) |
| Desmoplastic fibroma sinus maxillaris | 1 (16.7) | 2**^a^** | 1 (20.0) | 0 | 1 (100) | 0 | 1 (25.0) |
| Epidermal cyst in ear canal | 1 (16.7) | NA | 1 (20.0) | 0 | 0 | 0 | 1 (25.0) |
| Laryngomalacia | 1 (16.7) | ≤2**^a^** | 1 (20.0) | 0 | 0 | 0 | 1 (25.0) |
| Submandibular gland tumor | 1 (16.7) | 49**^a^** | 1 (20.0) | 0 | 0 | 0 | 0 |
| Unspecified tumor of nasal cavity | 1 (16.7) | 2**^a^** | 1 (20.0) | 0 | 0 | 0 | 0 |
| Endocrine system |  |  |  |  |  |  |  |
| Pancreas | 20 (5.2) | 26 | 9 (5.7) | 11 (4.9) | 3 (7.7) | 9 (7.3) | 8 (40.0) |
| Parathyroid | 6 (1.6) | 43.0 | 3 (1.9) | 3 (1.3) | 1 (2.6) | 3 (3.4) | 4 (66.7) |
| Pituitary | 5 (1.3) | 22.5 | 1 (0.6) | 4 (1.8) | 0 | 1 (0.8) | 1 (20.0) |
| Thyroid | 29 (7.6) | 37 | 5 (3.1) | 24 (10.8) | 5 (12.8) | 6 (54.5) | 11 (37.9) |
| Adrenal angiomyolipoma | 17 (4.5) | 22.5 | 6 (3.8) | 11 (4.9) | 0 | 3 (2.4) | 5 (29.4) |
| Heart | 4 (1.0) | 27.0 | 1 (0.6) | 3 (1.3) | 2 (5.1) | 1 (0.8) | 1 (25.0) |
| Cardiac lipoma | 3 (75.0) | 28 | 1 (100) | 2 (66.7) | 2 (5.1) | 1 (0.8) | 0 |
| PEComa, heart | 1 (25.0) | 2 | 0 | 1 (33.3) | 0 | 0 | 1 (100) |
| Intestinal |  |  |  |  |  |  |  |
| Bile duct and gall bladder | 4 (1.0) | 31.5 | 3 (1.9) | 1 (0.4) | 0 | 1 (0.8) | 0 |
| Duodenum | 2 (0.5) | 11 | 2 (1.3) | 0 | 0 | 2 (1.6) | 0 |
| Stomach | 3 (0.8) | 25 | 2 (1.3) | 1 (0.4) | 0 | 2 (1.6) | 0 |
| Liver | 19 (5) | 26 | 7 (4.4) | 12 (5.4) | 2 (5.1) | 8 (6.5) | 0 |
| Cysts | 9 (47.4) | 42 | 3 (42.9) | 6 (50.0) | 1 (50.0) | 3 (37.5) | 0 |
| Angiomyolipoma | 6 (31.6) | 29 | 1 (14.3) | 5 (41.7) | 0 | 3 (37.5) | 0 |
| Hamartoma | 3 (15.8) | 12 | 2 (28.6) | 1 (8.3) | 0 | 1 (12.5) | 0 |
| Hemangioma | 2 (10.5) | 31 | 2 (28.6) | 0 | 1 (50.0) | 1 (12.5) | 0 |
| Eye | 2 (0.5) | <1 | 0 | 2 (100.0) | 0 | 1 (100.0) | 0 |
| Bilateral anterior polar cataract | 1 (50.0) | ≤1^a^ | 0 | 1 (100) | 0 | 0 | 0 |
| Coloboma of iris | 1 (50.0) | ≤1^a^ | 1 (100) | 0 | 0 | 1 (100) | 0 |
| Lymphatic tissues | 9 (2.4) | 21 | 3 (1.9) | 6 (2.7) | 1 (2.6) | 6 (4.8) | 5 (55.6) |
| Chyloperitoneum lymphangioma | 1 (11.1) | 31^a^ | 0 | 1 (16.7) | 0 | 0 | 1 (100) |
| Lymph nodes from the renal resection region with pattern of angiomyolipoma | 1 (11.1) | 17^a^ | 0 | 1 (16.7) | 0 | 1 (16.7) | 0 |
| Lymphedema | 7 (77.8) | 21 | 3 (100) | 4 (66.7) | 1 (2.6) | 5 (83.3) | 4 (57.1) |
| Nervous system | 4 (1.0) | 19.5 | 2 (1.3) | 2 (0.9) | 0 | 3 (2.4) | 1 (25.0) |
| Arachnoid cyst | 2 (50.0) | 22.5 | 1 (50.0) | 1 (50.0) | 0 | 1 (33.3) | 1 (100) |
| Perineural cyst | 1 (25.0) | 28a | 0 | 1 (50.0) | 0 | 1 (33.3) | 0 |
| Syringomyelia | 1 (25.0) | 11 | 1 (50.0) | 0 | 0 | 1 (33.3) | 0 |
| Other | 6 (1.6) | 18.5 | 2 (1.3) | 4 (1.8) | 0 | 2 (1.6) | 2 (33.3) |
| Angiomyolipoma, other localization | 3 (50.0) | 24 | 2 (100) | 1 (25.0) | 0 | 1 (50.0) | 1 (100) |
| Abdominal cystic pelvic tumor | 1 (16.7) | 13^a^ | 0 | 1 (25.0) | 0 | 0 | 1 (100) |
| Hamartoma, left breast | 1 (16.7) | 10^a^ | 0 | 1 (25.0) | 0 | 1 (50.0) | 0 |
| Lung tumor | 1 (16.7) | NA | 0 | 1 (25.0) | 0 | 0 | 0 |
| Skeletal, soft tissue | 264 (69.1) | 24 | 118 (74.2) | 146 (65.5) | 23 (59.0) | 87 (70.2) | 31 (11.8) |
| Bone sclerotic foci | 151 (57.2) | 31.5 | 59 (50.0) | 92 (62.3) | 14 (35.9) | 44 (50.6) | 3 (2.0) |
| Scoliosis | 88 (33.3) | 13 | 45 (38.1) | 43 (29.5) | 6 (15.4) | 33 (37.9) | 20 (22.7) |
| Hemihypertrophy | 12 (4.5) | 4.5 | 5 (4.2) | 7 (4.8) | 0 | 5 (5.7) | 1 (8.3) |
| Calvarium sclerosis and thickening | 4 (1.5) | 14 | 2 (1.7) | 2 (1.4) | 0 | 2 (2.3) | 0 |
| Chordoma | 3 (1.1) | 3 | 3 (2.5) | 0 | 1 (2.6) | 1 (1.1) | 3 (100) |
| Hip dysplasia | 2 (0.8) | 33 | 0 | 2 (1.4) | 0 | 1 (1.1) | 0 |
| Cartilaginous exostosis | 1 (0.4) | 11^a^ | 0 | 1 (0.7) | 1 (2.6) | 0 | 0 |
| Club foot | 1 (0.4) | 6^a^ | 0 | 1 (0.7) | 0 | 0 | 1 (100) |
| Fibrolipoma of upper limb | 1 (0.4) | 8^a^ | 1 (0.8) | 0 | 0 | 1 (1.1) | 0 |
| Fibrous hamartoma T2 spine | 1 (0.4) | 1 ^a^ | 0 | 1 (0.7) | 0 | 0 | 0 |
| Fibrous bone disorder | 1 (0.4) | missing | 0 | 1 (0.7) | 0 | 0 | 0 |
| Foot inversion | 1 (0.4) | 8 ^a^ | 1 (0.8) | 0 | 0 | 0 | 1 (100) |
| Hernia diaphragm | 1 (0.4) | 33 ^a^ | 0 | 1 (0.7) | 0 | 0 | 0 |
| Kyphosis | 1 (0.4) | 4^a^ | 1 (0.8) | 0 | 0 | 0 | 0 |
| Myositis ossificans | 1 (0.4) | 2^a^ | 0 | 1 (0.7) | 0 | 1 (1.1) | 0 |
| Neurofibroma, mandible | 1 (0.4) | 15 | 1 (0.8) | 0 | 0 | 0 | 1 (100) |
| Osteochondrome | 1 (0.4) | 3^a^ | 1 (0.8) | 0 | 0 | 0 | 1 (100) |
| Plantar fibromatosis | 1 (0.4) | 21^a^ | 0 | 1 (0.7) | 0 | 0 | 0 |
| Spina bifida occulta | 1 (0.4) | 7^a^ | 1 (0.8) | 0 | 1 (2.6) | 0 | 0 |
| Unspecified neoplasm in thoracic region | 1 (0.4) | 2^a^ | 1 (0.8) | 0 | 0 | 0 | 0 |
| Spleen  Angiomyolipoma | 9 (2.4)  9 (100) | 13 | 4 (2.5) | 5 (2.2) | 1 (2.6) | 4 (3.2) | 3 (33.3) |
| Urogenital |  |  |  |  |  |  |  |
| Ovary | 9 (2.4) | 16 | 0 | 9 (4.0) | 2 (5.1) | 5 (71.4) | 1 (11.1) |
| Ovarian cyst | 8 (88.9) | 16 | 0 | 8 (88.9) | 2 (100) | 4 (80.0) | 1 (12.5) |
| Ovarian tumor | 1 (11.1) | 34^a^ | 0 | 1 (11.1) | 0 | 1 (20.0) | 0 |
| Bladder  Lyoma | 1 (0.3)  1 (100) | 43 | 1 (0.6) | 0 | 0 | 0 | 0 |
| Urethra  Hypospadias | 2 (0.5)  2 (100) | <1 | 2 (1.3) | 0 | 0 | 2 (1.6) | 0 |
| Uterus | 3 (0.8) | 45 | 0 | 3 (1.3) | 0 | 2 (1.6) | 2 (66.7) |
| PEComa | 2 (66.7 | 30.5 | 0 | 2 (66.7) | 0 | 1 (50.0) | 2 (100) |
| Myoma | 1 (33.3) | 54^a^ | 0 | 1 (33.3) | 0 | 1 (50.0) | 0 |

^a^Actual age at diagnosis.

NA, not applicable; PEComa, perivascular epithelioid cell neoplasms.

**Supplementary Table 2. Rare manifestations and the rationale for their exclusion from the analysis**

| Manifestations captured in case report form as rare manifestations | Reason for exclusion |
| --- | --- |
| Juvenile angiofibroma | **Major TSC manifestation or clinical sequelae of a major manifestation** |
| Extracardiac rhabdomyoma, anterior to right ventricle |  |
| Renal angiomyolipoma |  |
| Neurofibroma right wrist |  |
| Epilepsy |  |
| Symptomatic epilepsy |  |
| Subclinical epilepsy |  |
| Generalized epilepsy |  |
| Epilepsy controlled with treatment |  |
| Nodular change on the right cheek |  |
| Drug-resistant epilepsy |  |
| Retinal astrocytoma |  |
| Cortical dysplasia |  |
| Hypomelanotic areas |  |
| Facial angiokeratoma |  |
| Polycystic kidney disease | **Minor TSC manifestation** |
| Kidney cystic tumor |  |
| Left renal cyst |  |
| Nothing | **Mentioned as ‘nothing’, ‘none’ or ‘no rare manifestation’** |
| None |  |
| No rare manifestation |  |
| Strabismus | **No clear rare manifestation or clinical symptom with several potential reasons** |
| Hearing decrease |  |
| Paresis of tongue and nasolabial folds |  |
| Hepatosplenomegaly |  |
| Asymmetric face (left>right) |  |
| Hypothyroid |  |
| Astigmatism |  |
| Amblyopia |  |
| Plaque left scapulae |  |
| Hydrocephalus |  |
| Right hemiplegia |  |
| Knee cysts |  |
| Chronic constipation |  |
| Tumor brain |  |
| Nephrolith |  |
| Dizziness |  |
| Stomatitis, migraines, epistaxis |  |
| Stones in gallbladder |  |
| Shortness of breath, fat deposits in legs and edema in stomach |  |
| Macrodystrophia lipomatosa, back |  |
| Kidney tubers |  |
| Cervical adenitis |  |
| Hypertrophy tonsils |  |
| Voluminous tonsils |  |
| Right hemiparesis |  |
| Blindness following a binocular intracranial hypertension |  |
| IgG deficiency |  |
| Failure to thrive |  |
| Hydronephrosis |  |
| Talus valgus bilateral, surgical procedure |  |
| Hepatomegaly |  |
| Mild splenomegaly |  |
| Severe mental retardation (IQ <30), language retardation |  |
| Renal microcalcificates |  |
| Small renal hyperechogenity |  |
| Fracture dens axis (costa 2) secondary to seizure/fall |  |
| Reduced sense of hearing, left side |  |
| Paroxysmal changes in EEG but no epilepsy |  |
| Double kidney right with vesicorenal reflux |  |
| Thrombocytopathy |  |
| Subcutaneous ganglioma on wrist |  |
| Left buttocks mass | **Not evaluable due to unclear description** |
| Cystic degeneration |  |
| Abnormality of the pancreas, details unknown |  |
| Scalp raised lesion (benign) |  |
| Hypoplasia |  |
| After abdominal peritoneal high echo |  |
| Pelvic tumor |  |
| Polypoid lesion |  |
| A hard mass over right breast |  |
| Small mediastinal lymph nodes |  |
| Lymph nodes |  |
| Mild sacral dimple, simple |  |
| Adenoma |  |
| Astrocytoma |  |
| Glial tumor with hemorrhage and sequelar hemiparesis |  |
| Entrapment in the pelvis |  |
| Dorsal lipoma |  |
| Uterine lesion |  |
| Lipoma |  |
| Asymmetric buttocks |  |

EEG, electroencephalogram; IgG; immunoglobulin G; IQ, intelligence quotient; TSC, tuberous sclerosis complex.
